# Editorial: Global excellence in infectious diseases – Surveillance, prevention and treatment: Central and South America

**DOI:** 10.3389/fmed.2022.1084753

**Published:** 2022-11-28

**Authors:** Olivia Valenzuela, Isadora C. de Siqueira

**Affiliations:** ^1^Departamento de Ciencias Químico Biológicas, Universidad de Sonora, Hermosillo, Mexico; ^2^Instituto Gonçalo Moniz, Fundação Oswaldo Cruz, Ministério da Saúde, Salvador, Brazil

**Keywords:** Central and South America, SARS-CoV-2, influenza A virus, acquired immunodeficiency syndrome, Chagas disease, dengue, Guillain-Barré syndrome, leptospirosis

Infectious diseases affecting humanity continue challenging the scientific community and the health sector worldwide; several bacteria, viruses, fungi, and parasites can be classified into this group. The emergence of severe acute respiratory syndrome coronavirus 2 (SARS-CoV-2), responsible for coronavirus disease 2019 (COVID-19), demonstrated that control and prevention of infectious diseases require the sum of global efforts and community participation. Despite international efforts, COVID-19 has provoked over 200,000 deaths monthly globally in the last 2 years. Hospitalizations, long-term COVID-19 deaths, and work absences are the main COVID-19-associated factors responsible for economic depression. Low- and middle-income countries, such as Africa and Latin America, have been significantly affected. Therefore, it is important to maintain the molecular diagnosis of SARS-CoV-2 in the population, independent of symptom presentation. This strategy will provide an opportunity to identify new variants (Kuriyama et al.). In addition, this Research Topic presents preliminary data suggesting that nimotuzumab (anti-EGFR) is safe and can reduce mortality in cases of severe and critical COVID-19 (Diaz et al.). Influenza A virus is another important pathogen because it provokes respiratory infections worldwide and causes annual epidemics. In addition, the virus can potentially cause pandemics (such as H1N1, 2009). Influenza A produces an acute respiratory and febrile illness that is generally self-limiting in healthy people. However, a group of individuals is highly susceptible, such as children, pregnant women, elderly individuals, immunocompromised individuals, and underlying chronic disease patients. In this group of patients, influenza can cause death (das Chagas Sousa et al.). Obesity and its comorbidities, such as diabetes, have been classified as a non-infectious pandemic. Importantly, they generate the main complications in various infectious diseases, such as influenza.

Acquired immunodeficiency syndrome (AIDS) is another significant infectious disease affecting people worldwide. AIDS, caused by human immunodeficiency virus (HIV), provoking an estimated 1.5 million (1.0–2.0 million) new cases annually, and an esteemed 38 million people globally are infected with HIV. Exist risk factors associated with the HIV prevalence higher, include men who have sex with men, intravenous drug users, people in prisons, and sex workers. Due to antiretroviral therapy (ART), a considerable reduction in the morbidity and mortality associated with HIV has been noticed; however, many undiagnosed cases provoke a continued transmission of HIV and an increase in morbimortality. In the case of low-middle-income countries, other factors have been associated with this phenomenon, such as loss of clinical follow-up, treatment withdrawal, and rising resistance to some drugs (Quirola-Amores et al.).

Diseases transmitted by vectors affect many countries in Latin America, such as those transmitted by mosquitos (mainly by *Aedes aegypti*) and triatomines (family Reduviidae). Dengue virus (DENV) has four serotypes (DENV-1,−2,−3, and−4) and was considered among the “top 10 threats to global health” in 2019 by the World Health Organization. Dengue is the leading cause of death and illness among individuals infected with an arbovirus. It is estimated that almost half of the world's population is at risk of Dengue infection. Annually, 390 million new cases are reported, and nearly 100 million patients are affected with varying disease severity. In the case of Latin America and the Caribbean, 3.1 million cases were reported in 2019, the highest ever. Regarding the serotypes, the evidence showed that DENV-1, -2, -3, and -4 co-circulated in Brazil, Guatemala, and Mexico, and the number of severe dengue exceeded reports in the preceding 4 years. As occurs in other infections, children usually have the most severe presentations of dengue and the highest imputable morbidity and mortality (May Lue et al.). Guillain-Barré syndrome and its atypical variant, Miller-Fisher syndrome, can be associated with viral infections. The Research Topic presents a brief research report describing the association of these syndromes with Zika, Chikungunya, and Dengue viruses (Santana do Rosário et al.). Chagas disease, also known as American trypanosomiasis, was recognized by the World Health Organization as a neglected tropical disease (2005), provoked by a hemoflagellate protozoan, *Trypanosoma cruzi*. This parasite is transmitted predominantly through the feces or urine of triatomine insect vectors infected but also through congenital, transfusions, transplants, laboratory accidents, and oral routes. Is a potentially life-threatening illness, affecting over 6 million people in the Americas, and 7,500 deaths are associated with Chagas annually; Although endemic of 21 continental Latin American countries it has become a global disease. The control of Chagas is challenging mainly because the parasite has high genetic variability, as well as the great diversity of reservoirs of the parasite *T. cruzi* (wild animals such as pets) and a broad biodiversity of triatomine vectors. Unfortunately, there is no accurate standard assay for the serologic diagnosis of chronic *T. cruzi* infection. Given this situation, the recommendation of WHO and PAHO is to use two serologic tests to improve diagnosis. Another problem is that diagnosis procedures vary by location (endemic or non-endemic areas) and in the screening of blood/organ donors. In addition, the genetic polymorphism of this protozoan affects the test's performance and the geographic region where the screening tests are performed. A critical observation is that over 20% of new cases worldwide are provoked in blood banks, where people become infected with Chagas through contaminated blood transfusions. These results highlight the importance of universal donor screening to exclude Chagas disease. One alternative is using chimeric IBMP antigens to decrease the number of bags discarded due to false-positive results (Ferreira dos Santos et al.).

Leptospirosis, a reemerging disease, is a worldwide zoonosis caused by spirochetes of the genus Leptospira. This Research Topic includes a manuscript that evaluates the humoral response and the factors involved in the exposition of leptospirosis. This work assessed the antibody response of individuals with a natural and asymptomatic infection and compared the results of a biannual and quarterly serological survey (Cruz et al.).

Metagenomic next-generation sequencing is an essential diagnostic tool to identify uncommon pathogens. A case report on this Research Topic used metagenomic next-generation sequencing on cerebrospinal fluid analysis to diagnose anaerobic meningitis (Li et al.).

Many people living with infectious diseases are socioeconomically vulnerable and have limited access to medical care and limited access to diagnosis, treatment, and vaccines. Therefore, infectious disease surveillance (routine testing) must be a priority in all countries for the early detection of new infection outbreaks. The cost-utility analyses are essential because they provide crucial information and, according to Quirola-Amores et al., must be considered throughout the establishment, restructuring, implementation, or maintenance of a strategy for the control and prevention of diseases. In summary, the articles on this Research Topic discussed Surveillance, Prevention, and Treatment of important infectious diseases in Central and South America.

## Author contributions

All authors listed have made a substantial, direct, and intellectual contribution to the work and approved it for publication.

## Conflict of interest

The authors declare that the research was conducted in the absence of any commercial or financial relationships that could be construed as a potential conflict of interest.

## Publisher's note

All claims expressed in this article are solely those of the authors and do not necessarily represent those of their affiliated organizations, or those of the publisher, the editors and the reviewers. Any product that may be evaluated in this article, or claim that may be made by its manufacturer, is not guaranteed or endorsed by the publisher.

